# Editorial: F-18 FDG PET/CT Imaging: Normal Variants, Pitfalls and Artifacts

**DOI:** 10.3389/fnume.2022.965615

**Published:** 2022-07-15

**Authors:** Jasna M. Mihailovic, Ronan P. Killeen

**Affiliations:** ^1^Faculty of Medicine, University of Novi Sad, Novi Sad, Serbia; ^2^Department of Radiology, St. Vincent's University Hospital, Dublin, Ireland; ^3^School of Medicine, University College Dublin, Dublin, Ireland

**Keywords:** F-18 FDG-PET/CT, Ga-68 DOTATATE PET/CT, Ga-68 PSMA PET/CT, normal variants, pitfalls, artifacts

F-18 FDG-PET/CT is a hybrid imaging technology that includes computed tomography (CT) and positron emission tomography (PET) and thus provides anatomical (morphologic) and functional (metabolic) data at the same time. Nowadays, F-18 FDG-PET/CT is most frequently used in oncology, but due to non-specific F-18 FDG uptake it can also be used for imaging of benign disorders such as infections and inflammatory disorders. F-18 FDG is the most commonly used radiotracer. In addition, Ga-68 radiotracers were recently introduced for imaging of prostate cancer and neuroendocrine tumors (Ga-68 PSMA and Ga-68 DOTATATE/DOTATOC, respectively).

Normal variants, artifacts, and incidental findings are common on PET/CT imaging. Thorough knowledge of normal anatomy, basic physiology, image acquisition, attenuation correction artifacts, patients' history, false negatives, and false positives is important to maximize diagnostic accuracy and patient outcome.

In this Research Topic, we have seven articles on the topic written by a group from Johannesburg, University of Witwatersrand, in South Africa. They have discussed the normal variants, pitfalls, and artifacts of PET/CT imaging in patients with malignant and benign diseases, with a special focus on F-18 FDG PET/CT, Ga-68 PSMA PET/CT, and Ga-68 DOTATATE PET/CT. The authors provide an excellent collection of PET/CT images and clinical examples for each article.

F-18 FDG PET/CT is not specific for cancer imaging and may also be used for the detection of infective and inflammatory diseases. Mbakaza and Willy Vangu provide a nice overview of musculoskeletal, infective, and inflammatory uptake including normal variants, pitfalls, and artifacts on F-18 FDG PET/CT imaging. Their analysis of cases will be useful for the interpretation of PET/CT scans to aid the differentiation between infection/or inflammation and malignant tissue in oncologic patients.

F-18 FDG-PET/CT plays an important role in staging and re-staging of high-risk melanoma (AJCC Stages III and IV). In addition, in patients with cutaneous melanoma PET/CT is useful for initial staging, evaluation of treatment response, recurrence detection, and prognosis of the disease. Momodu and Vangu discuss the different biological and molecular characteristics of various melanoma types that may be associated with imaging pitfalls. They discuss normal variants on PET/CT imaging that include normal F-18 FDG uptake in benign disorders not related to malignant disease. Potential false positive findings evident on PET/CT are outlined including brown adipose tissue activation, trauma, and intramuscular injection site. Of interest, the authors note that patients with retroviral disease often have reactive lymph nodes that demonstrate increased FDG avidity. These avid lymph nodes may be difficult to differentiate from malignant disease, particularly in patients with lymphoma or cervical cancer and underlying HIV.

F-18 FDG-PET/CT imaging has great value in lymphoma patients, not only in initial staging of disease but also in evaluation of treatment response and prognosis. Vangu and Momodu(a) give a nice overview of PET/CT imaging in lymphoma with common normal physiological variants, technical artifacts, and diagnostic pitfalls. Brown fat activation is particularly problematic in interpretation of nodal involvement in patients with lymphoma or head and neck cancer. The authors show excellent images of misregistration artifact, attenuation correction artifact that may also lead to misdiagnosis. Additionally, the authors provide a nice collection of images in various treatment settings, such as posttreatment thymic hyperplasia in young patients and bone marrow activation.

F-18 FDG-PET/CT imaging is often used for the evaluation of abdomen and pelvic malignancy. Benign disorders may demonstrate increased FDG uptake that may mimic malignant pathology. In addition, technical artifacts that can occur due to metallic hardware, motion, truncation and effects of contrast agents may compromise accurate diagnosis. Vangu and Momodu(b) discuss the PET/CT normal physiologic variants, artifacts and pitfalls including pictorial illustrations with excellent examples of false positive and false negative findings.

Physiologic distribution of FDG in children is different than in adults. Purbhoo and Vangu outline why optimal image interpretation requires comprehensive knowledge of normal anatomy, physiologic FDG distribution uptake, normal variants, pitfalls, and artifacts of PET/CT imaging in pediatric oncology. Finally, the authors emphasize radiation safety in children. Techniques for dose reduction and modified PET/CT acquisition protocols used to decrease radiation dose but without compromising diagnostic information in children are outlined.

Due to the low avidity of F18-FDG in most prostate cancer cells, F18-FDG PET/CT has a low sensitivity for prostate cancer. In contrast, PSMA PET/CT has a significant value in the diagnostic algorithm of prostate carcinoma, in particular in staging of high-risk prostate cancer patients and detection of biochemical recurrence. Furthermore, based on theranostic approach PSMA PET/CT also helps in selection of patients with metastatic castrate resistant prostate cancer for treatment with Lu-177. However, PSMA expression is not specific to prostate cancer and may accumulate in various non-prostate cancer diseases with PSMA expression. PSMA uptake has been reported, for example, in benign bone disorders (fractures, osteomyelitis, multiple myeloma, and Paget's disease) and, inflammatory conditions (tuberculosis, sarcoidosis, and diverticulosis). Malan and Vangu(a) discuss PSMA normal biodistribution, its uptake in non-prostate cancer disorders and artifacts on PET/CT imaging. They have provided excellent illustrations of clinical examples and a table with teaching points. They highlight the role of comprehensive knowledge of physiologic PSMA distribution, and medical history to improve diagnostic accuracy.

Ga-68 DOTA compounds are used for well-differentiated neuroendocrine tumors in staging and restaging. In addition, based on theranostic approach they can also be used for patient selection for radionuclide therapy. Malan and Vangu(b) have shown several clinical examples of potential pitfalls and artifacts. Finally, they outline that comprehensive knowledge of normal Ga-68 DOTA distribution, image acquisition, false positives and false negatives are required for accurate PET/CT interpretation. In addition, the authors outline the role of additional imaging modalities such as MRI and CT in patient assessment.

We thank our colleagues from South Africa for their contribution and comprehensive reviews with illustrations on the topic. We also thank the editors of Frontiers who gave us the opportunity to present this topic to our readers.

## Author contributions

All authors listed have made a substantial, direct, and intellectual contribution to the work and approved it for publication.

## Conflict of interest

The authors declare that the research was conducted in the absence of any commercial or financial relationships that could be construed as a potential conflict of interest.

## Publisher's note

All claims expressed in this article are solely those of the authors and do not necessarily represent those of their affiliated organizations, or those of the publisher, the editors and the reviewers. Any product that may be evaluated in this article, or claim that may be made by its manufacturer, is not guaranteed or endorsed by the publisher.

